# Sexual assault: women’s voices on the health impacts of not being believed by police

**DOI:** 10.1186/s12905-021-01358-6

**Published:** 2021-05-22

**Authors:** Karen McQueen, Jodie Murphy-Oikonen, Ainsley Miller, Lori Chambers

**Affiliations:** 1 School of Nursing, Lakehead University, 955 Oliver Rd, Thunder Bay, ON P7B5E1 Canada; 2 School of Social Work, Lakehead University, Thunder Bay, Canada; 3 Lakehead University, Gender and Women’s Studies, Thunder Bay, Canada

**Keywords:** Sexual assault, Women’s voices, Unfounded sexual assault, Health and social impact, Police response, Canada

## Abstract

**Background:**

Sexual assault is a prevalent crime against women globally with known negative effects on health. Recent media reports in Canada indicate that many sexual assault reports are not believed by police. Negative reporting experiences of sexual assault have been associated with secondary victimization and trauma among survivors. However, little is known about the impact that being sexually assaulted and not believed by police has on a survivor’s health and well-being. The purpose of this study was to explore women’s experiences of not being believed by police after sexual assault and their perceived impact on health.

**Methods:**

We conducted open-ended and semi-structured interviews with 23 sexual assault survivors who were sexually assaulted and not believed by police. The interviews explored the self-reported health impacts of not being believed by police and were conducted from April to July, 2019. All interviews were audio-recorded, transcribed, and entered into NVIVO for analysis. Data were analyzed using Colaizzi’s analytic method.

**Results:**

Analysis revealed three salient themes regarding the health and social impact of not being believed by police on survivors of sexual assault: (1) Broken Expectations which resulted in loss of trust and secondary victimization, (2) Loss of Self, and (3) Cumulative Health and Social Effects. The findings showed that not being believed by police resulted in additional mental and social burdens beyond that of the sexual assault. Many survivors felt further victimized by police at a time when they needed support, leading to the use taking of alcohol and/or drugs as a coping strategy.

**Conclusion:**

Reporting a sexual assault and not being believed by police has negative health outcomes for survivors. Improving the disclosure experience is needed to mitigate the negative health and social impacts and promote healing. This is important for police, health, and social service providers who receive sexual assault disclosures and may be able to positively influence the reporting experience and overall health effects.

**Supplementary Information:**

The online version contains supplementary material available at 10.1186/s12905-021-01358-6.

## Background

The high rate of sexual violence against women is a concerning public health issue as approximately one in three women in North America experience sexual assault in their lifetime [1, 2]. While sexual assault is a pervasive social issue that does not discriminate against age, gender, ability or status, research indicates that certain groups are at greater risk [1, 3, 4]. This includes women who are of colour [1], Indigenous, [3, 5, 6], employed in the military, living and working in underprivileged environments [3], have a disability [7], and student populations [3, 8].

Sexual assault, defined as any type of forced or coerced sexual contact or behavior that happens without consent [9], violates the sexual integrity of individuals and exposes them to a variety of negative health outcomes [10]. These may include, but are not limited to social, psychological [10–13], sexual [12–14], and physical health outcomes [15–17] that may have lifelong deleterious effects on survivors.

In North America, police response to sexual assault has been highly criticized based on a culture of victim blaming and stereotyping which result in disbelief of sexual assault reports [18, 19]. Rape myths also perpetuate the belief that many women lie about assault [4, 20] and that rape only occurs to women who choose to live risky or chaotic lifestyles [21]. Recent Canadian media reports indicate that these societal myths are abundant in law enforcement, as a high number of sexual assault reports are not validated, and many sexual assault cases have been classified as “unfounded” [22]. According to the criminal code of Canada, when a case is classified as “unfounded” by the police, it is determined that a crime neither occurred nor was it attempted in the first place [23]. This contrasts with sexual assaults classified as unsubstantiated, as this classification is reserved for cases in which evidence is lacking and validation of the crime cannot be determined [23]. As such, the code “unfounded” is indicative of the victim not being believed and interpreted as lying. However, a meta-analysis of 7 studies found that the actual rate of false reporting (e.g., lying) about sexual assaults was low (approximately 5%) and typically reflected mental health concerns, misunderstanding of what constitutes sexual assault, and altered memory due to drug and alcohol use [24]. The findings from the meta-analysis suggest that many cases are inappropriately labelled as unfounded or not believed by police. A positive step was recently taken to remove minimize the use of the term “unfounded” from through expanded Canadian crime reporting option statistics; however, the change in codes does not automatically translate into increased belief of women’s sexual assault reports to the police. While the term unfounded is specific to Canadian reporting, evidence exists that not being believed by police is a widespread issue and not unique to Canada [25, 26].

The experience of sexual assault and not being believed by police has not been explored from women’s first-hand accounts. Research has identified that many sexual assault survivors have had negative reporting experiences with police that can negatively impact well-being [27]. Other studies report that negative experiences may lead to secondary victimization [28] or trauma [29, 30]. Given the known negative health effects that sexual assault has on survivors, coupled with the potential detrimental effects of a negative reporting experience, there is a need for a better understanding of how not being believed by police impacts women’s health. This is important for police, health, and social service providers who work with women who have been sexually assaulted, as a multidisciplinary response [31] to sexual assault is needed across the health, social, and legal sectors. The guiding principles of a multi-disciplinary approach are to ensure the survivors are safe, that their voices are heard, and that they have the autonomy to decide what they need for healing [32]. Thus, the purpose of this study was to understand the health impacts for women who were sexually assaulted and not believed by the police. For our study, health was broadly defined as a state of physical, mental and social well-being [33].

## Methods

### Study design

The study involved qualitative research and explored the lived experience of women who were sexually assaulted and not believed by the police [34]. It particularly explored the self-reported health impacts of study participants. It used a phenomenological approach to capture the lived experiences of participants [35]. The consolidated criteria for reporting qualitative research (COREQ) guided the conduct of this study [36].

### Sampling and recruitment of participants

Purposive sampling was used to recruit a sample of participants from a large geographic area in northwestern Ontario, Canada, between April 13th and July 21st, 2019. Recruitment strategies included social media advertising (e.g., Facebook, Twitter, and Instagram) and through word of mouth from social service providers who had been informed of the research. Individuals wishing to learn more about the research study were invited to contact a member of the research team via cellular telephone or email. During the initial contact, the research process was explained and potential participants were invited to ask questions.

The eligibility criteria to participate in the study included: (a) being an English-speaking female 16 years of age or older, having experienced a sexual assault, which was reported to police, (b) self-report that the sexual assault was not believed by the police, and (c) ability to provide consent and participate in interviews within the designated study region. Participants were excluded from the research if: (a) the police laid charges and/or the perpetrator was taken to court, (b) the survivor of the sexual assault did not wish to pursue charges, or (c) the survivor self-identified that they were unsure whether a sexual assault occurred (unclear events or memories). A research assistant screened prospective participants by telephone to ensure they met the inclusion criteria for the research. The Principal Investigator or Co-Investigator provided a full explanation of the study, re-assessed eligibility and obtained informed consent before commencing the interview. Written consent was obtained for all in-person interviews. Two participants who were interviewed through telephone provided verbal consent. The informed consent process included providing information on the purpose of the research, risks and benefits of participation, anticipated outcomes and dissemination of the information gathered through the research. All participants were informed of their rights including not answering questions, asking for clarification, requesting a break and/or ending the interview at any time. All participants who provided written consent were provided with a copy. The consent process (written and verbal) was approved by the research ethics board.

Thirty-seven women expressed interest in the research. Twenty-three women met the inclusion criteria of having experienced a sexual assault, reported the assault to the police, and perceived that they were not believed by police. Participant’s perceptions of police disbelief were determined by various factors such as the short duration of police report with little or no note taking, location of the reporting (not at the police station), no incident numbers provided, blaming questions, lack of a thorough investigation, no follow-up on case outcomes from the police, no charges laid, and/or no return phone calls from the police. Some participants indicated that police explicitly stated that they did not believe the woman’s report. One participant provided a copy of the police report, which confirmed that the police did not believe her report. There were no individuals who refused to participate in the study; however, the researchers excluded thirteen transcripts from the analyses based on the exclusion criteria. Despite reviewing inclusion and exclusion criteria prior to the interview, it emerged during some interviews that the women were believed by police. In three cases police laid charges and the perpetrators went to court and in six cases the women chose not to pursue charges. Two of the sexual assault cases were not reported to the police and two self-identified that they suspected but were not certain a sexual assault had occurred (e.g., they stated that “something didn’t feel right”, but had no recollection of a sexual assault). One participant was excluded as she was unable to provide consent due to being intoxicated.

#### Data collection

Data collection was conducted by an all-female research team and included one to two semi-structured face-to-face (n = 21) or telephone interviews (n = 2) based on participant preference. Second interviews were conducted with two participants based on their request to provide additional information. Interviews were conducted in the University campus and led primarily by the principal investigator or lead author with expertise in interviewing and qualitative methods. A second member of the research team was present to collect demographic data, take field notes and support the interview process. There were no other individuals present during the interview. Prior to commencement of the interview, introductions were made and the purpose and rationale for the research were outlined for the participant, allowing an opportunity for questions to be addressed.

The interviews were open-ended and followed a semi-structured interview guide developed for this study. The interview guide was informed by the literature and researcher expertise in relation to the purpose of the study (see Additional file 1). To isolate the impact of not being believed from that of the sexual assault, women were asked to describe the impact on health (if any) when their sexual assault report was not believed by the police and how that differed from the impact of the sexual assault on their health and well-being. In addition to the interviews, a demographic questionnaire was completed by participants after the interviews. The average length of the interviews was 53 min. A second interview occurred on two occasions when participants called back to report information that they felt was important and not included in the first interview. The research team discussed termination of conducting further interviews based on saturation of the data. A few additional interviews were conducted and included in the analysis once saturation had been achieved since they had been previously scheduled.

Following each interview, participants were provided with an honorarium as a token of appreciation for their time. In addition, all participants were provided with a list of support services available in the community if they felt they required additional support. The university institutional research ethics board (ref # 1466856) approved all study related materials (e.g., consent, recruitment advertisement, and interview guide).

### Data analysis

To aid in the analysis, all interviews were audio recorded and transcribed verbatim. All interviews were conducted in English, which was the language of the participants, hence no translation of the transcripts was required. Transcripts were entered into NVIVO version 11. Colaizzi's [37] analytic method was used as a means of organizing and analyzing the data. This approach included extensive reading of transcribed data, extraction of significant phrases, a constant comparative method, and a comprehensive thematic description of the accounts of participants by three study team members (JM-O, KM, AM). A fourth study team member (LC) reviewed all themes and data collected within each theme to verify the findings. Discussion occurred between all (n = 4) research team members to ensure the interpretation of findings was accurate; no changes to the original thematic analysis were deemed necessary. All participants were invited through email to validate themes. Five participants requested to review the thematic findings. These findings were discussed via telephone with the principal investigator. The five participants confirmed the findings and did not add any further information.

## Results

### Participant characteristics

Of the twenty-three women who participated in the study, approximately half self-identified as Caucasian (n = 12; 52%) or Indigenous (n = 11; 48%). The average age of women at the time of the study was 37 years (range 22 to 57 years). Most women reported being single (n = 15; 65%), unemployed (n = 13; 56%), and earning an annual income less than $19,999 (n = 16; 69%). Participants had various levels of education, including partial high school (n = 6; 26%), high school completion (n = 1; 4%), college (n = 9; 39%), and university (n = 5; 22%), while two did not report their levels of education.

The circumstances of the sexual assault were mixed. More than half of the women (n = 15; 65%) reported knowing the perpetrators (intimate partner [n = 1], family member [n = 3], acquaintance/friend [n = 11]), while eight (35%) of the perpetrators were strangers. Likewise, the response to the sexual assault varied, with nine (39%) reporting that they physically and/or verbally resisted the attacker (n = 11; 48%). Drugs and alcohol were a factor in many of the sexual assaults, with 57% (n = 13) of perpetrators having consumed alcohol, nine of whom, had also used drugs. For the assaults by strangers, women were unsure whether the perpetrator had used drugs or alcohol. Among survivors, half reported (n = 12; 52.2%) using substances prior to the assault. Of these, the majority used alcohol only (n = 8), alcohol and drugs (n = 3) or drugs only (n = 1). Three additional participants reported that they were forcibly given alcohol and/or drugs prior to the assault. Five participants (21.7%) suspected that they were drugged by the perpetrator prior to being sexually assaulted.

Analysis of the interview transcripts revealed three salient themes related to the health and social impact of not being believed by the police. These included: (a) Broken Expectations, (b) Loss of Self, and (c) Cumulative Health and Social Effects*.* Within the theme of Broken Expectations, two subthemes emerged including Loss of Trust and Secondary Victimization*.*

### Broken expectations

Overall, most participants noted that the purpose of the police was to protect the public. They disclosed that this sense of safety and protection contributed to their sense of well-being and was disrupted during their report of sexual assault. Accordingly, when the women made their sexual assault reports to police, they believed that the police would assist and investigate their complaints. However, the participants explained that there was incongruence between what they believed would happen when they reported and what actually occurred. They described feelings of disappointment, being let down, having an additional pain, or that their sexual assault reports fell on “*deaf ears*” (033). “*They are supposed to be there to help you and keep you safe, and I didn’t think they were doing it at all*” (010)*.* An Indigenous participant who reported her sexual assault to the police and waited more than 24 h for a police response, described how she thought making a report would be the first step of the healing process after the sexual assault, but believed that her experience with the police made it [sexual assault experience] all worse:

You think that they [police] are going to protect you, right, and that they are going to do justice. What happened had happened [sexual assault] and that couldn’t be undone. I don’t know how to explain it, like kind of a step towards healing, if that makes sense. Like that would have been my first step, instead it just kind of made a new pain to have to deal with. (009)

Participants’ expectations were broken when the police did not act and investigate their complaints. Before reporting, participants believed that the police would take their complaints seriously. Participants reported that they expected “*I would get called back*” (008), “*that the assault would be investigated*” (001) or “*they would take my report*” (022). However, many said that they never heard back from police and that their concerns were never followed up. These broken expectations led to adverse social and health outcomes for participants when they perceived they were not being believed. This included loss of trust in the police and secondary victimization by the police.

#### Loss of trust

Many participants reported experiencing loss of trust in the police and the justice system after not being believed and/or their report not being followed-up. Much of the discussion regarding loss of trust was based on participants’ perceptions that the police did not care about them as individuals or that the police had too many other things to deal with. This loss of trust led women to feel that there was no point in seeking assistance from the police in other situations. A young woman who was sexually assaulted while sitting alone in a secluded area, reported the assault to the police, and recalled that the police laughed at her when she disclosed that she was assaulted by a stranger. Their response precipitated her lack of trust; “*We will take care of each other, because they* [police] *don’t care about us and…you know, they don’t believe you anyways, so don’t even bother. We will deal with our own stuff*” (012)*.* Another participant who was sexually assaulted and reported the assault to the police following a medical examination at the hospital, described feeling interrogated by the police, with the police officer’s initial question being “*Did you just do it and regret it*” (017)? This experience led to her lack of trust and subsequent protective instincts with her two young children, “*I always tell my kids police are safe to go to, but there is still hesitation with that…I wouldn’t discourage them from it, but I would rather have them come to me and then I’ll help them if something ever happened*” (017)*.*

The loss of trust in police impacted some women who experienced further violence or sexual assaults and did not make subsequent reports out of fear of not being believed by the police. An Indigenous participant who reported feeling immense shame when the police did not believe that her sexual assault had occurred, described the impact of the police response on her subsequent experience with violence, “*When I first got into my domestic* (i.e. intimate partner abuse)*, the relationship was violent, like I never called* [the police]*, I never called anytime that I got hit. I never felt like they would believe me anyway*” (009)*.* Similarly, despite being drugged during her sexual assault, and left “hogtied” (i.e. hands and feet tied together) on a street, a participant who perceived her involvement in the sex trade contributed to not being believed by the police, described the impact of the disbelief on her well-being, “*Well, I was in a relationship where I was beat, beat very badly, and it took me 2 years before I reported it…because they wouldn’t believe me*” (029)*.* Participants’ prior experience with police greatly impacted their subsequent physical and mental health, as several indicated enduring subsequent abuse instead of reporting to the police.

#### Secondary victimization

Women had various motivations to report their experiences of sexual assaults to the police. Many of the women wanted to report their experiences to the police in order to have the perpetrator charged, or to prevent others from being assaulted. Some indicated that they felt nervous to report to the police and only did so with encouragement from family, friends or healthcare/service providers. When the women did report, and were not believed, some felt being further victimized by the reporting experience and the questions they were asked. A young woman who was sexually assaulted after a social outing with friends on a University campus, described her interaction with the police, thus:

His [police officer] demeanour, like a bit of everything; his tone. I remember his tone because he was like well did you do this? Even the way he asked it was like, what were you wearing? … That’s the one thing I can really remember, and I said those questions shouldn’t stick with me for the rest of my life, but they do. I feel like sometimes that was worse than the actual incident (017).

Similarly, another participant who was involved in sex work, commented that the police response to her report was “*well you work the streets, you bring it on yourself*”*.* Furthermore, the police officer stated, “*serves you right for being out there on the corners and out at night, you women ask for it. I will never forget that; you women ask for it*” (029)! The experience of being let down by the professional who is supposed to protect you was perceived as emotionally damaging well beyond the trauma of the sexual assault. It is this disbelief from the police that left women feeling defeated, unsafe and unworthy of protection and support.

#### Loss of self

All participants reported that not being believed by police impacted them at a personal level. Many indicated that the disbelief by police impacted their well-being and made them feel as if a part of them had been lost or taken away. The women described feelings of loss regarding their self-worth, self-esteem, self-image, and/or self-confidence after not being believed. A woman who was drugged by her perpetrator, had a rape kit done at the hospital, reported the assault to the police and felt dismissed by them, discussed the impact of disbelief from the police on her sense of self, thus:

It took away from who I was as a person before that, and it chipped away at my self-esteem. It made me think ok, well if these people think that I am not worthy of investigation and not worthy of fighting for, then why should I fight for myself? Why should I fight for my life (022)?

Similarly, another participant who was sexually assaulted repeatedly by her step-brother, explained the impact that the disbelief from the police had on her self-worth, stating, “*You feel like if the police don’t care what happens to you, why should I, right*” (033)? The internalization of the police response was further articulated as, “*I do the same thing that they did to me, I sweep me under the carpet*” (i.e. ignore my own needs) (033)*.* The narratives by participants show that many of the women felt that the lack of investigation and validation of their report was synonymous with not being worthy or important enough. The lack of validation made one woman view herself as “*a lying, drunk piece of shit really*” (018)*.*

Women also reported that not being believed impacted how they perceived themselves or how they were perceived by others. They indicated that they felt “*guilty*”, “*like a liar*”, “*like I did something wrong*”*, *“*dismissed*”*, *“*shame*” and “*angry*” after not being believed. Some women reported that they blamed themselves or felt guilty like it was their fault for going to the bar the night they were assaulted or that they willingly went with the individual who later assaulted them. Despite being the victims of crime, the interaction that women had with the police prompted an internalization of shame and personal responsibility for something outside of their control.

### Cumulative health and social effects

Participants reported that the sexual assault negatively impacted their health. All (n = 23, 100%) women self-reported negative effects on their mental health including Post Traumatic Stress Disorder (PTSD), depression, and escalation or initiation of alcohol or drug abuse. Thirteen (57%) had physical injuries (e.g., bruises, cuts, head injury) and many reported negative social effects including subsequent homelessness (n = 10, 31%) and/or future unreported assaults (sexual or physical) (n = 17, 74%). At times, it was difficult for the women to specifically separate out the effects of the sexual assault from those of not being believed by police on their health and well-being. However, participants reported that not being believed by police exacerbated or resulted in additional negative effects on their overall health and well-being. Many indicated that the experience of being sexually assaulted and then not being believed had a cumulative effect, which made the impact worse. One participant who was sexually assaulted in high school stated that,

I became very angry at the world that I had to go through that and nobody else did and or no one else even cared or believed me. It was a very dark place in my life. I tried to commit suicide after that too at one point. I had a lot of mental health issues (033).

Women used various descriptors in their narratives to explain how not being believed by police had both significant and long-lasting health and social effects. In terms of the magnitude of the impact, a woman who was sexually assaulted by a relative reported that not being believed “*probably affected me the worst*” (009). Another indicated that “*it did have an effect on me emotionally, very strongly*” (001) and “*you know I felt like that* [emotional effects] *for a very long time*” (022). Not being believed by police also meant that there would never be closure of the cases and this left women to wonder what could have been, or how their lives might have been different. A woman who experienced sexual assault from a distant family member explained the impact of the lack of closure of her case, “*who would I be if these things never happened to me? Or maybe if the police believed me and there was closure to the case, would that make a big difference to me or would it not? I don’t know*” (033). Similarly, a participant who found the courage to report a sexual assault away from her home environment in another community, had her file transferred numerous times without any police officer taking full responsibility. She explained that not being believed added to the sexual assault, “*I think it’s the whole thing, the big picture, the whole experience. You know, like why me? What did I do to deserve this?* (033)”*.*

## Discussion

This study explored the impact that being sexually assaulted and not believed by the police had on women’s health. Overall, women reported that not being believed by police had a negative impact on their mental (e.g., low self-esteem, loss of self-worth, secondary victimization) and social health (e.g., loss of trust, experiencing and not reporting further assaults) beyond the impact of the sexual assault (e.g., PTSD, substance use, physical injuries etc.). This indicates that not being believed by police resulted in additional mental and social burdens during an already traumatic period. For many of the women, they perceived the impact to be both significant and long-lasting. These findings suggest that women who were not believed by police felt further victimized and may have suffered additional trauma.

Our findings are consistent with the general sexual assault literature that suggests that women often experience secondary victimization from lack of investigation, insensitivity, and perceived judgment from police officers, as well as low rates of arrests and sentencing of the perpetrator(s) [38]. Unexpected negative reactions, including blame, judgment, disbelief, and lack of empathy may negatively influence well-being and recovery [39, 40]. Negative social reactions to sexual assault disclosures may amplify feelings of powerlessness, grief, loss, and disenfranchisement [13], shame [41, 42], increased severity and duration of PTSD [43] and other mental health concerns [44]. When the sense of self (i.e. self-worth) is negative, there are often greater risk-taking behaviours as survivors may not feel that they are worthy of protection or deserving of safety and wellness [43]. The women in our study, who had negative reporting experiences, lost trust in police, and reported experiencing further assaults and victimization. Additionally, as women were not believed by police, they were often not referred for support or treatment, leaving survivors to either attempt to heal from within, or turn to health and social service providers for support on their own. Without treatment, women felt that the negative effects of the sexual assault and not being believed were cumulative, severe and long lasting. Furthermore, many had poor mental health (e.g., PTSD, depression, anxiety) and reported escalation of substance use as a coping mechanism.

Women’s first-hand accounts of their reporting experiences from our study highlight the associated trauma that presents when women’s reports are not believed by police. When women’s sexual assault disclosures are responded to with empathy and validation, they experience less adverse health outcomes [45]. In addition to improved health, women who have positive experiences are also likely to utilize adaptive coping strategies such as cognitive restructuring, expressing emotion and meditation [46] and are likely to approach formal assistance networks (e.g., mental health or primary care provider) [47]. Given the deleterious effects of not being believed by the police on the health and well-being of survivors, and the known benefits of an empathic response, health and social service professionals may be well positioned to assist women in rebuilding their lives, promoting positive coping strategies, and ensuring that women’s voices matter.

Health and social service professionals can reduce the impact of secondary victimization [48] and the negative effect on health and well-being of survivors by believing survivors’ accounts. Health and social service providers should determine the experiences women had with police in order to ensure that survivors are provided with a safe environment and their dignity is maintained. Person-centered care is central to assisting survivors of sexual assault heal from their experiences. Thus, providing compassionate care and preserving the dignity and well-being of the survivor [49] is essential for mitigating the impact of the assault and the experience of disbelief from the institutions designated to protect them. Comprehensive care for survivors is needed for immediate and long-term health benefits [50]. Professionals are also well-equipped to fulfill an advocacy role with sexual assault survivors [32] which may assist in re-visiting the sexual assault report, safety planning, achieving justice through law enforcement, and subsequently assisting in closure of cases for survivors.

Our research has demonstrated that sexual assault survivors fear reporting subsequent victimization to the police due to a lack of trust in them. Fear of reporting is problematic as access to positive formal support may mitigate the severity of trauma reaction among survivors [39]. Health and social service providers trained in trauma informed care can increase sexual assault reporting by supporting survivors and advocating for sensitive treatment from the police. While numerous factors may impact women’s recovery from the trauma of sexual assault [51], enhancing the disclosure experience is one strategy that may be beneficial for attaining long-term health and wellbeing of survivors, given that sexual assault is already one of the most under-reported crimes [52].

The lack of reporting of sexual assault is problematic for several reasons. First, underreporting of sexual assault underestimates the severity of the problem and results in under-estimation of the number of individuals affected. Having an accurate recording of the extent of sexual violence is necessary for the allocation of resources for preventive and treatment services [53]. Safety is also of concern, as recidivism of sexual perpetration is a risk for both the survivor and/or other individual(s). Furthermore, if women do not disclose the sexual assault, they are likely to suffer in silence and are at risk of missing out on supportive services. Given that survivors of trauma often feel betrayed and may have difficulty trusting others [54], their reluctance to report is not surprising. The findings of this study support the need to provide a safe space for survivors to disclose sexual violence without being further victimized [54].

### Implications for policy and practice

The women in our study articulated the negative impact that not being believed by police had on their well-being beyond the sexual assault. Their voices (e.g., research themes) need to be highlighted to provide a better understanding of women’s experiences, which may lead to increased sexual assault reporting and improved response from law enforcement.

The large number of Indigenous women in our sample requires further exploration. Rates of sexual violence against Indigenous women are three times greater compared to other groups [3], and historical patterns of maltreatment and being dismissed by police still persist [55–57]. Their experiences may be unique and require targeted interventions (i.e. focus on cultural safety) to meet their needs.

Sensitization regarding women’s experiences and how to respond positively to survivors is required for community service providers, police, and healthcare professionals who receive sexual assault reports [26, 44, 58]. Sensitivity training for police officers that incorporates women’s voices and experiences can also serve to reduce bias, and improve belief of survivors. This may be particularly important in small communities where there are no specialized units to investigate sexual assaults. Strategies that survivors feel are helpful include providing time to talk about their experiences (i.e. listening), expressing belief in their experiences, telling them it is not their fault, and promoting agency [44]. Avoiding blame, offering support, validating their experiences, providing support and follow-up [43] and safety planning are also important for survivors’ health and well-being [44]. Furthermore, incorporating trauma-informed principles such as establishing safety, respect, choice, collaboration, and empowerment are additional strategies that could facilitate a positive disclosure experience and minimize the possibility of re-traumatization [59]. Using this type of approach recognizes the impact violence has on individuals and minimizes secondary victimization [59]. Information, education and communication campaigns are also needed to challenge the normalization of rape culture myths, so that victims feel safe to report and/or seek services for sexual assault [60].

## Limitations

Participation is the study was voluntary and limited to women who self-disclosed that their sexual assault report to police was not believed. We were unable to determine whether the cases were classified as unfounded by police. This study presents only the perspective of those who perceived that they were not believed and did not include those who reported sexual assault and had positive experiences. Although participants included a diverse sample of women (i.e. by ethnicity, education, and socio-economic status) the study design precluded comparison within the sample. The participants in this study were primarily women from low socio-economic background with limited education residing in one geographic area and the findings may not be generalizable to all women.

## Conclusions

Sexual assault has long been associated with adverse outcomes for survivors. Not being believed by police after reporting a sexual assault has an additional negative impact on survivors beyond that of the assault. The findings of this study suggest that efforts are needed to improve the support for sexual assault disclosures for women so that they can report sexual assault without experiencing further victimization, thereby increasing their safety and potentially mitigating secondary victimization. The women’s voices from our study may provide police and health and social service providers with a better understanding of the experiences of sexual assault survivors thereby improving police response.

## Supplementary Information

**Additional file 1.** Interview Guide.

## Data Availability

Data used in this paper are not publicly available due to the sensitive nature of the topic and the risk of breaching confidentiality. The data are available on reasonable request from the lead author.

## Abbreviations

COREQ: Consolidated criteria for reporting qualitative research
PTSD: Post-traumatic stress disorder
