# Correction to: Sexual assault: women’s voices on the health impacts of not being believed by police

**DOI:** 10.1186/s12905-021-01380-8

**Published:** 2021-06-03

**Authors:** Karen McQueen, Jodie Murphy-Oikonen, Ainsley Miller, Lori Chambers

**Affiliations:** 1 School of Nursing, Lakehead University, 955 Oliver Rd, Thunder Bay, ON P7B5E1 Canada; 2 School of Social Work, Lakehead University, Thunder Bay, Canada; 3 Gender and Women’s Studies, Lakehead University, Thunder Bay, Canada

## Correction to: BMC Women’s Health (2021) 21:217 10.1186/s12905-021-01358-6

Following the publication of the original article [1], we were notified that few words were not deleted within one revised sentence on page 2, 2nd paragraph:

- “A positive step was recently taken to remove minimize the use of the term “unfounded” from through expanded Canadian crime reporting option statistics”.
- Should read: “A positive step was recently taken to minimize the use of the term "unfounded" through expanded Canadian crime reporting options. “

The original article has been corrected.
