# Supplementary material for: Sexual assault: women’s voices on the health impacts of not being believed by police
Source: BMC Womens Health. 2021 May 22;21:217. doi: 10.1186/s12905-021-01358-6 (PMC8141152; doi:10.1186/s12905-021-01358-6)
Supplement: Supplementary file 1 — Additional file 1. Interview Guide. [file 12905_2021_1358_MOESM1_ESM.docx]

**Supplementary File 1**

**Interview Guide: Unfounded Sexual Assault**

Can you tell me why you decided to participate in this research? Do you have any concerns about the interview?

As you know, this research is exploring women’s experiences with sexual assault, particularly when their sexual assault was not believed by police. Can you tell me about the sexual assault?

- what happened

- where

- when

- type of SA

After you were sexually assaulted, did you speak to anyone about what happened?

- Why or why not?
- How did you decide who you would speak to?
- How did the person you spoke to react to your disclosure?

**Reporting**

How did you make the decision to contact the police to report the sexual assault?

What was your motivation for reporting the sexual assault to the police?

Ie. Justice, protect others

When you reported the sexual assault to the police, what did you expect would happen?

Did you have concerns about reporting your sexual assault to the police? Please explain.

How has the experience of reporting the sexual assault to the police impacted your life?

Have you had previous experience with the police? If yes, how does this experience compare?

Do you recall your emotional state or the way you presented at the time of the report?

What do you recall about your interaction with the police when you were making a report that you were sexually assaulted?

-ie police response, types of questions, how you felt etc.

Did you ever feel like your behavior was being questioned? If yes, how did this impact you?

Were you ever “warned” that you could be charged with false reporting? Please explain.

Did you ever feel pressured to drop your case? Please explain.

When you reported the sexual assault to the police, was support offered to you? Did you speak to anyone?

**Decision to Unfound the Sexual Assault**

Were you informed by the police that your assault was deemed “unfounded” or that they did not believe you?

- Can you recall what was communicated to you from the police?
- How did they communicate this to you? (Phone, in person, etc.)
- Did they explain their rationale for the decision?

When you learned that your sexual assault case was deemed unfounded or not believed, how did you feel about the decision?

- How did it impact you?

Why do you believe that police did not believe you that you were sexually assaulted?

When you reported the sexual assault to the police, did you have any support in the community? Please explain.

-prompt ie. Crisis worker, counsellor etc.

**Post-unfounded Decision**

How did you feel when you learned that the police did not believe you?

When the sexual assault was deemed unfounded or not believed, who did you talk to? Did this decision by the police impact your personal relationships in any way? Please explain.

Did the decision about the sexual assault impact your faith in the justice system? Would you be likely to report sexual assault to the police if it occurred in future?

If a friend or family member experienced a sexual assault, would you advise them to report to the police? Can you explain your position?

- Did the support that you received meet your needs? Please explain.

**Impact on Health**

Have you experienced any health concerns since your sexual assault that you believe are connected to the assault? Ie. Mental health, physical health or sexual health

Have you been formally diagnosed by a healthcare provider with any type of physical or mental health problem since you were sexually assaulted?

Do you feel that the disbelief about the sexual assault from the police affected your health in any way? Please explain.

- Difficulty sleeping, eating, etc.

**General Questions Re: Police**

What would you like to see changed in the future in terms of police response to sexual assault?

Is there anything you were asked about during the investigation of the sexual assault that bothered you?

In a perfect world, what type of police response would have been most helpful to you?

Did you ever change your mind about pursuing charges? If so, what prompted you to change your mind?
